# 904. Impact of a Pharmacist-driven Antimicrobial Stewardship Program on Prescribing Practices by Intensive Care Physicians in a Latin American Hospital.

**DOI:** 10.1093/ofid/ofac492.749

**Published:** 2022-12-15

**Authors:** José Pablo Díaz-Madriz, Esteban Zavaleta-Monestel, José Miguel Chaverri-Fernandez, Sebastián Arguedas-Chacón, Raquel Arguedas-Herrera, Brayan Leiva-Montero, Ana Fernanda Vásquez-Mendoza, Gabriel Muñoz-Gutierrez

**Affiliations:** Clínica Bíblica Hospital, San José, San Jose, Costa Rica; Pharmacy Director, Clínica Bíblica Hospital, San José, San Jose, Costa Rica; Associate Professor, University of Costa Rica, San José, San Jose, Costa Rica; Pharmacy Intern, University of Costa Rica, San José, San Jose, Costa Rica; Pharmacy Intern, University of Costa Rica, San José, San Jose, Costa Rica; Pharmacy Intern, University of Costa Rica, San José, San Jose, Costa Rica; Bacteriologist, Clínica Bíblica Hospital, San José, San Jose, Costa Rica; Clínica Bíblica Hospital, San José, San Jose, Costa Rica

## Abstract

**Background:**

In a recent report from Latin American hospitals, only 59.7% of the prescriptions of patients admitted to the ICU followed treatment guidelines. Implementation of antimicrobial stewardship programs (AMS) have proven to be an effective tool for the rational use of antimicrobials, but their implementation has been a challenge in this region due to resources limitations. One significant barrier is the lack of clinical pharmacists with specialized training in infectious diseases. This study aims to determine the impact of a pharmacy driven AMS on the optimal prophylactic and empirical therapy prescription by intensive care physicians (ICP) in a Latin American hospital.

**Methods:**

A retrospective observational study that compared the optimal selection and the consumption (DOTs/1000 ICP patient days) of antibiotics in patients treated by intensive care physicians before and after the implementation of the AMS at Clínica Biblica Hospital, Costa Rica. The comparison was made from January to December 2014 (preAMS) and from January 2020 to March 2021 (postAMS). Bacterial resistance patterns before and after AMS implementation were also compared.

**Results:**

A total of 333 patients met the inclusion criteria (52% preAMS and 48% postAMS). After a five-year intervention, the optimal antibiotic selection prescribed by ICP was 43.1% (n=75) in the preAMS period and 86.8% (n=138) in the postAMS period (43.7% absolute improvement, p < 0.001). The prescription of most antibiotics showed an improvement (Fig 1), such as ertapenem in 45% (p< 0.001) and levofloxacin in 59% (p< 0.001). Similarly, there was a trend towards improvement of the prescription by diagnosis (Fig 2), including an increase of 32% (p< 0.001) in CAP. There was a reduction of the consumption of most antimicrobials (Fig 3) including a decrease of 66.9% (p =0.017) for vancomycin and 64.7% (p =0.033) for meropenem. Regarding bacterial resistance, there was found a decrease of 11% (p=0.048) of P. aeruginosa resistant to meropenem and a reduction in the detection of ESBL in E. coli (11% decrease; p=0.007).

**Figure:**
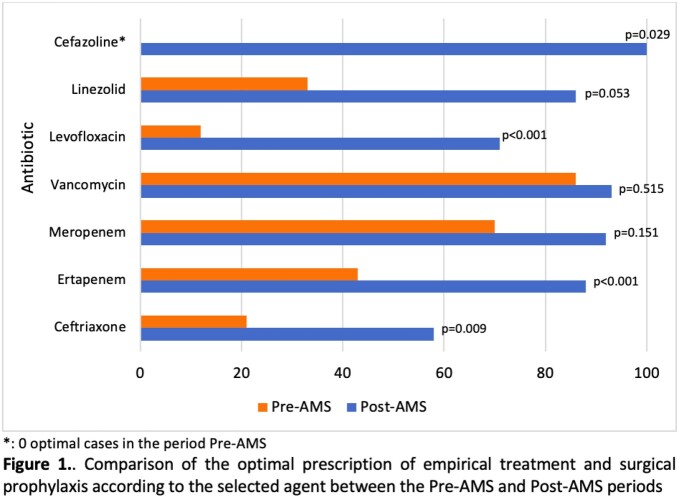

**Figure:**
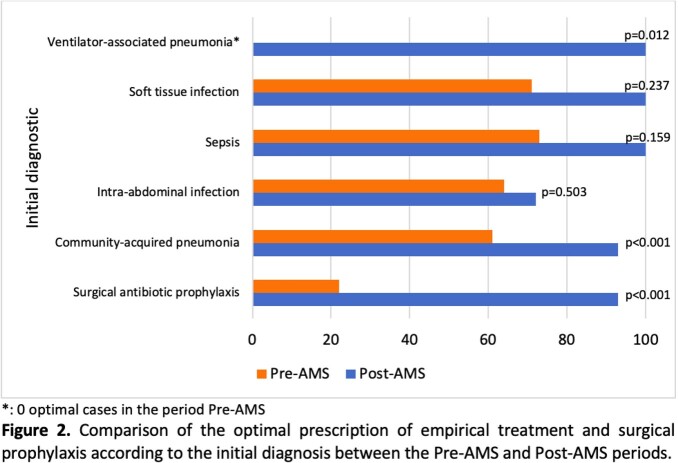

**Figure:**
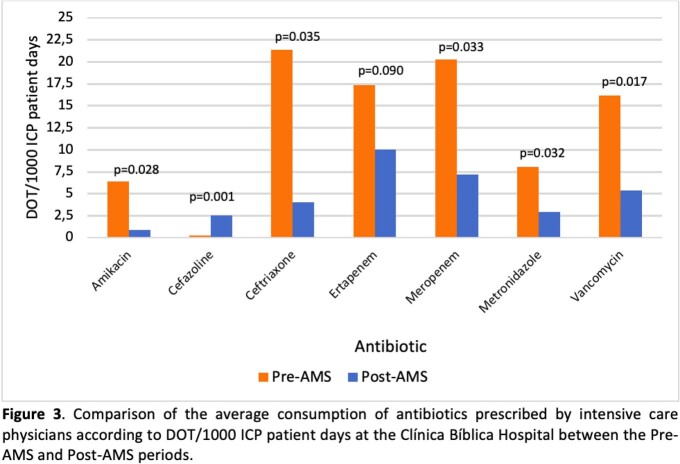

**Conclusion:**

The AMS implementation showed an overall positive impact on antibiotics selection and consumption. In addition, is thought that the intervention could had a positive effect on antibiotic resistance.

**Disclosures:**

**José Pablo Díaz-Madriz, PharmD, MSc**, Eli Lilly: Stocks/Bonds|Pfizer: Advisor/Consultant.

